# Cytotoxic Effects of the Synthetic Cannabinoid, 5F-MDMB-PICA on Human Glioblastoma U87-MG Cells

**DOI:** 10.7150/ijms.125987

**Published:** 2026-04-16

**Authors:** Mohammed Mufadhe Alanazi, Fawaz Alasmari, Mohammad M. Algahtani, Abdullah S. Alhamed, Khaled A. Alhosaini, Mohammed M. Almutairi, Faleh Alqahtani, Wael A. Alanazi, Faisal N. Alharbi, Raghad Alsanie, Mohammed Alshehri, Youssef Sari

**Affiliations:** 1 Department of Pharmacology and Toxicology, College of Pharmacy, King Saud University (KSU), Riyadh, Saudi Arabia.; 2 Department of Pharmacology and Experimental Therapeutics, College of Pharmacy and Pharmaceutical Sciences, University of Toledo, Toledo, OH 43606, USA.

**Keywords:** 5F-MDMB-PICA, U87-MG, neurotoxicity, inflammation, oxidative stress, mitochondrial dysfunction

## Abstract

Synthetic cannabinoids, particularly 5F-MDMB-PICA, have emerged as potent psychoactive substances, exhibiting a binding affinity to the CB1 receptor approximately 380 times greater than that of Δ9-THC, the principal psychoactive compound in cannabis. Their widespread abuse has been associated with severe intoxication and fatalities globally. This study aimed to investigate the cytotoxic effects and mechanisms of 5F-MDMB-PICA in U87-MG astrocyte-like cells, a common model in neurobiological research.

U87-MG cells (passages 7-15) were treated with increasing concentrations of 5F-MDMB-PICA (1-200 μM) for 24 hours. We employed several assays to evaluate cytotoxicity (MTT assay), oxidative stress, mitochondrial membrane potential, cellular migration (wound healing), and cell death (flow cytometry). Additionally, gene expression analysis via qRT-PCR focused on apoptotic and inflammatory markers. A receptor docking study was performed to verify similarities in binding sites between 5F-MDMB-PICA and Δ9-THC.

Our results showed that exposure to 5F-MDMB-PICA led to a concentration-dependent increase in cytotoxicity and oxidative stress, alongside a significant decrease in mitochondrial membrane potential. There was a marked elevation in apoptosis and necrosis, coupled with impaired cellular migration. Gene expression analysis revealed significant upregulation of pro-apoptotic and inflammatory genes, such as P53, Bax, TNF-α, and COX-2, indicating a robust pro-apoptotic and pro-inflammatory response. Notably, key residues (Phe200 and Ser383) in the CB1 binding pocket were identified as crucial for the interaction of both compounds, which might provide insight into the shared patterns of cytotoxic effects.

These findings highlight the neurotoxic effects of 5F-MDMB-PICA, underscoring the serious public health risks associated with synthetic cannabinoids.

## 1. Introduction

**Figure 1:**
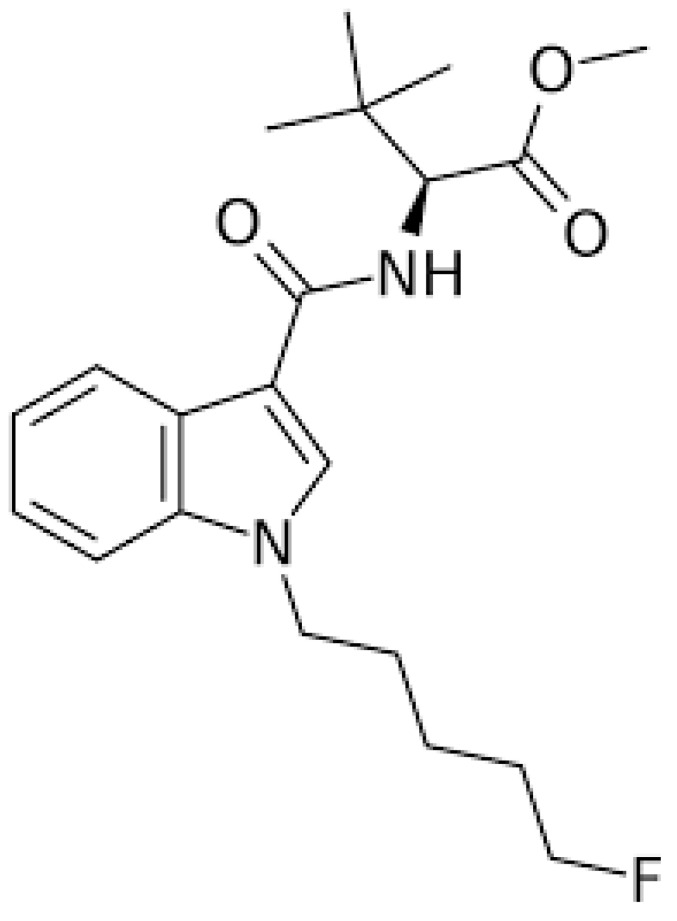
** The Chemical Structure of 5F-MDMB-PICA.** methyl 2-(1-(5-fluoropentyl)-1H-indole-3-carboxamido)-3,3-dimethylbutanoate.

Synthetic cannabinoids (SCs) are active substances that mimic the effects of tetrahydrocannabinol (THC), the main psychoactive substance in cannabis, that interact with cannabinoid receptors 1 (CB1) and 2 (CB2). THC acts as a partial agonist of these receptors, whereas synthetic cannabinoids act as full agonists of both CB1 and CB2. This indicates that they have greater affinity and higher potency for CB1 receptors than THC. Consequently, these compounds can induce considerably stronger harmful effects and exhibit severe toxicity 1. Cannabinoid receptors are G-protein-coupled receptors 2. It has been reported that CB1 receptors are primarily expressed in the central nervous system (CNS), whereas CB2 receptors are primarily expressed in the peripheral systems, particularly the immune system 3. Historically, SCs were designed, synthesized, and developed in the 1980s to study the effects of endogenous cannabinoids on the brain. They have been linked to aggression, hallucinations, changes in perception and mood, memory impairment, panic attacks, difficulties with concentration, confusion, dizziness, psychosis, slurred speech, and even coma 4. Furthermore, research indicates that cannabinoids can trigger apoptosis, neuroinflammation, excitotoxicity, and oxidative stress in both *in vitro* and *in vivo* experimental models 5. For example, Chen et al. have shown that selected SCs can inhibit proliferation and influence both apoptosis and ferroptosis in cellular models 6. Another study found that certain SCs, such as CUMYL-4CN-BINACA, can trigger apoptosis, generate reactive oxygen species, and induce endoplasmic reticulum stress and inflammation in neuronal tissues 7. Dogan et al. demonstrated that the SC, Cumyl-4CN-BINACA, induces oxidative stress and activates apoptotic and inflammatory pathways 8. Additionally, a recent study confirmed that indolic SCs exhibit cytotoxic and antiproliferative effects 9. Interestingly, a recent study found that both original synthetic compounds (SCs) and their pyrolysis byproducts are classified as neurotoxic substances. These compounds exert their effects by triggering excitotoxicity and activating neuroinflammatory and oxidative stress pathways 10. SCs are a rapidly growing category of psychoactive substances initially described in patent applications between 2009 and 2011 11. Banister et al. evaluated the structure-activity relationships of this subclass of synthetic cannabinoids and synthesized various compounds bearing valine or tert-leucine amide substituents, as well as their methyl ester analogs 11. 5F-MDMB-PICA (methyl 2-(1-(5-fluoropentyl)-1H-indole-3-carboxamido)-3,3-dimethylbutanoate) (Fig. 1) is a synthetic cannabinoid that is classified as a Schedule I controlled substance by the DEA (Drug Enforcement Administration). It demonstrated the highest potency within this group, with an activity 380 times greater than that of Δ9-THC, the main psychoactive compound in natural cannabis. This compound acts via the human cannabinoid receptor subtype (CB1) 11 and is considered the indole analog of the well-known synthetic cannabinoid 5F-ADB (methyl N-{[1-(5-fluoropentyl)-1H-indazol-3-yl] carbonyl}-3-methylvalinate). It is commonly found in “legal high” products, such as herbal smoking mixtures 12,13, vape liquids, or paper products infused with the substance for smuggling into prisons 13,14. The use of 5F-MDMB-PICA is associated with serious intoxication, overdose, and even increased risk of death 13,15. It is considered a full agonist that binds to and activates the CB1 receptor, with a high affinity and potency 13. Activation of this receptor by 5F-MDMB-PICA is associated with agitation, mood swings, confusion, logorrhea, behavioral changes, anxiety, and compulsive-like conditions 15,16. However, the effects of 5F-MDMB-PICA are not limited to the brain. Exposure to this SC can lead to cardiovascular complications, such as increased heart rate, shortness of breath, and chest pain. In severe cases, SCs can have life-threatening effects on the cardiovascular system, including suppression of cardiac and respiratory functions, circulatory collapse, and cardiac arrest 17. Owing to the adverse effects of 5F-MDMB-PICA, the United States Drug Enforcement Administration (DEA) placed the substance under emergency Schedule I control in 2019 18. This designation indicates that 5F-MDMB-PICA has a high potential for abuse and no accepted medical use, thereby restricting its legal manufacture, distribution, and possession. The widespread presence of 5F-MDMB-PICA in various “legal high” products, its association with serious toxicity and overdose, and its exceptionally high potency, underscore the significant public health threat posed by this synthetic cannabinoid. Thus, the emergency scheduling action of the DEA reflects the urgent need to regulate the availability of this potentially dangerous substance. The biological and toxicological data on the use of SCs are limited, owing to the rapid synthesis and production of these substances. In addition, the pharmacological and toxicological properties of SCs remain largely understudied because these compounds were discovered relatively recently. Therefore, this study aimed to evaluate the modulatory effects of 5F-MDMB-PICA on astrocyte-like U87-MG cells, focusing on cell viability, apoptosis, oxidative stress, mitochondrial function, and inflammation. Our findings may provide insights into the impact of this novel SC on astrocyte-like U87-MG cells and elucidate the mechanisms underlying the harmful effects of 5F-MDMB-PICA on the CNS.

## 2. Materials and Methods

### 2.1. 5F-MDMB-PICA Preparation

The SC 5F-MDMB-PICA was generously provided by Dr. Fawaz Alasmari. The compound was solubilized in dimethyl sulfoxide (DMSO) to create a stock solution, which is stored at -20 °C. Various concentrations of 5F-MDMB-PICA were subsequently prepared directly in the culture medium, as outlined in the cell culture section.

### 2.2. Cell Culture

U-87MG human glioblastoma cells (ATCC HTB-14; American Type Culture Collection, Manassas, VA, USA) were cultured between passages 5-9 at an initial density of ~3 × 10⁵ cells/mL in Dulbecco's Modified Eagle's Medium (DMEM; 1×, Gibco®, Grand Island, NY, USA) supplemented with 10% fetal bovine serum (FBS; South American origin, Gibco®) and 1% penicillin-streptomycin (100 U/mL and 100 μg/mL; Gibco®). Cells were maintained at 37 °C in a humidified 5% CO₂ incubator, with medium renewed every 48 h until reaching ~80-90% confluence. Experiments were conducted using cells at passages 7-15. For functional assays, cells were seeded into 96-well plates for cytotoxicity (MTT; 1 × 10⁴ cells/well, 24 h), reactive oxygen species (ROS; 3 × 10⁴ cells/well, 4 h), and mitochondrial membrane potential (JC-1; 2 × 10⁴ cells/well, 24 h) assessments. In parallel, 6-well plates were used for flow cytometry, polymerase chain reaction (PCR; 1 × 10⁶ cells/well, 24 h), and wound healing (scratch; 3 × 10⁵ cells/well, 24 h) assays. All treatments with 5F-MDMB-PICA were performed in complete culture medium containing FBS and penicillin-streptomycin.

### 2.3. Cytotoxicity Assay

Cytotoxicity was assessed using the MTT assay as previously described 14. Briefly, 3-(4,5-dimethylthiazol-2-yl)-2,5-diphenyltetrazolium bromide (MTT; MedChemExpress, USA) was employed to evaluate cell viability and mitochondrial metabolic activity. U-87MG cells were cultured, harvested, and trypsinized as described above, and then seeded into 96-well plates at a density of 1 × 10⁴ cells/well in 100 μL of complete medium. After 24 h of incubation, cells were treated with various concentrations of 5F-MDMB-PICA (1-200 μM) for an additional 24 h. Untreated cells served as the control group (CTRL). Following treatment, 10 μL of MTT solution (5 mg/mL in phosphate-buffered saline, PBS) was added to each well, and the plates were incubated for 1 h. The resulting formazan crystals were dissolved in 100 μL of dimethyl sulfoxide (DMSO) with gentle shaking for 5 min, and absorbance was subsequently measured at 570 nm using a microplate reader.

### 2.4. Oxidative Stress Assay

Intracellular ROS levels were quantified using an H2DCFDA kit (MedChemExpress, USA). This assay is based on the fluorogenic dye 2′,7′-dichlorofluorescin diacetate (H2DCFDA/DCF-DA), which passively diffuses into cells and is deacetylated by intracellular esterases to yield a non-fluorescent intermediate. Upon oxidation by intracellular ROS, this intermediate is converted to the highly fluorescent compound 2′,7′-dichlorofluorescein (DCF), which exhibits excitation and emission maxima at 485 and 529 nm, respectively. U-87MG cells were seeded in 96-well plates at a density of 3 × 10⁴ cells/well and allowed to adhere for 24 h. Cells were exposed to 200 μM 5F-MDMB-PICA for 4 hours, a concentration selected based on the MTT assay dose-response curve. Untreated cells were included as the control group (CTRL).

### 2.5. Mitochondrial Membrane Potential (Δψm- JC-1) Assay

Mitochondrial membrane potential was assessed using the JC-1 dye (MedChemExpress, USA), a well-established indicator of mitochondrial function. JC-1 exists in two forms: in healthy, non-apoptotic cells with intact mitochondrial membrane potential, it accumulates in the mitochondria as red-fluorescent aggregates, whereas in apoptotic or depolarized cells it remains in its monomeric green-fluorescent form. U-87MG cells were seeded into 96-well plates at a density of 2 × 10⁴ cells/well and allowed to adhere for 24 h. Cells were then exposed to 200 μM 5F-MDMB-PICA for 24 hours, a concentration selected based on the MTT assay dose-response curve. Untreated cells were included as the control group (CTRL). Changes in mitochondrial membrane potential were evaluated following the manufacturer's protocol.

### 2.6. Scratch Assay

The impact of 5F-MDMB-PICA on U-87MG cell migration was assessed using a scratch wound healing assay. U-87MG astrocyte-like cells were seeded and cultured to form a confluent monolayer by overnight incubation. The following day, a linear scratch was introduced into the monolayer using a sterile 200 μL pipette tip. Detached cells and debris were removed by replacing the medium, after which cells were treated with 5F-MDMB-PICA at concentrations of 50, 100, or 200 μM. Untreated cells served as the control group (CTRL). Images of the wound area were captured at 0 and 24 h using an inverted phase-contrast microscope equipped with a charge-coupled device (CCD) camera at 10× magnification.

### 2.7. Flow cytometry

Apoptotic and necrotic cell populations were quantified using annexin V-FITC/propidium iodide (PI) dual staining followed by flow cytometric analysis. U-87MG cells were seeded in 6-well plates at a density of 1 × 10⁶ cells/mL and incubated overnight to allow attachment. Cells were then exposed to 200 μM 5F-MDMB-PICA for 24 hours, a concentration selected based on the MTT assay dose-response curve. Untreated cells were included as the control group (CTRL). After treatment, cells were harvested by trypsinization, washed with phosphate-buffered saline (PBS) at 25 °C, and centrifuged at 1700 rpm for 5 min at 20 °C. The resulting pellet was resuspended in 400 μL of annexin V/PI binding buffer. An aliquot containing approximately 1 × 10⁵ cells in 100 μL was transferred into 1.5 mL microcentrifuge tubes, followed by the addition of 5 μL annexin V-FITC and 5 μL PI. Samples were gently mixed and incubated for 15 min at 25 °C in the dark. After incubation, 400 μL of 1× binding buffer was added to each tube, and the samples were analyzed within 1 h using a BD Accuri C6 flow cytometer. The proportion of viable cells was calculated by subtracting the percentage of annexin V-positive (apoptotic) and PI-positive (necrotic) cells from the total cell population.

### 2.8. Quantitative Real-Time Polymerase Chain Reaction

Total RNA was isolated from U-87MG astrocyte-like cells following treatment with 5F-MDMB-PICA at concentration of 200 μM for 24 h. Untreated cells served as the control group (CTRL). RNA extraction was performed using TRIzol reagent (Invitrogen/Life Technologies, Carlsbad, CA, USA) according to the manufacturer's protocol. Complementary DNA (cDNA) was synthesized from the extracted RNA using the Reverse Transcription Master Mix for qPCR (MedChemExpress, USA). Quantitative real-time PCR (qPCR) was subsequently carried out using Low ROX SYBR Green qPCR Master Mix (MedChemExpress, USA) and gene-specific forward and reverse primers targeting Bax, P53, COX-2, TNF-α, and the reference gene glyceraldehyde-3-phosphate dehydrogenase (GAPDH). Primers were obtained from Integrated DNA Technologies (Leuven, Belgium), and their sequences are provided in Table 1. Gene expression levels of Bax, P53, COX-2, and TNF-α were normalized to GAPDH expression, and relative mRNA levels were calculated using the 2^-ΔΔCT method.

### 2.9. Protein Preparation

The 3D coordinates of human cannabinoid receptor 1 (CB1) (PDB ID: 8GHV) was obtained for further analysis from Protein Data Bank (PDB) (https://www.pdb.org/pdb). This structure, resolved at 2.8 Å, was initially reported by Kumar et al. (2023) 19. To prepare the protein (chain D of the CB1) for molecular docking, all heteroatoms, including water molecules, ions, and any ligands, were systematically removed. Energy minimization of the pre-processed protein was per-formed using Chimera software version 1.13.1 (https://www.cgl.ucsf.edu/chimera/). This step is crucial to eliminate any unfavorable interactions that could interfere with molecular docking 20.

### 2.10. Preparation of Compounds for Molecular Docking against CB1 Receptor

PubChem, a comprehensive chemical database affiliated to the National Center for Biotechnology Information (NCBI) (https://cactus.nci.nih.gov/translate/), provided the 3D conformers for Δ9-Tetrahydrocannabinol (Δ9-THC) and 5F-MDMB-PICA. The structures of compounds were optimized using Open Babel (https://openbabel.org/index.html), an open-source program integrated into the Python Prescription environment. The optimization process utilized the Merck Molecular Force Field (MMFF94), which is well known for its precision in predicting the energies and geometry of organic molecules 21.

### 2.11. Molecular Docking of Δ9-THC and 5F-MDMB-PICA against CB1 Receptor

Molecular docking was performed using Auto Dock Vina to identify potential poses and orientations of compounds, along with their binding energies (BE) at the CB1 binding site 22. The molecular interaction fingerprints of the compounds were visualized using PyMOL© Molecular Graphics (version 2.4, 2016, Schrödinger LLC, New York, NY, USA) (Seeliger & De Groot, 2010) and BIOVIA's Discovery Studio (version 2016). This analysis highlighted key molecular interaction fingerprints, including hydrogen bonds, hydrophobic interactions, and electrostatic linkages between the CB1 amino acid residues and the ligand atoms.

### 2.12. Statistical Analyses

All data are expressed as the mean ± standard error of the mean (SEM). Statistical comparisons were conducted using one-way analysis of variance (ANOVA) followed by Tukey's post hoc test. A *P* value < 0.05 was considered statistically significant. All analyses were performed using GraphPad Prism software (version 8; GraphPad Software, CA, USA).

## 3. Results

### 3.1. 5F-MDMB-PICA-Induced U87-MG Cytotoxicity

**Figure 2:**
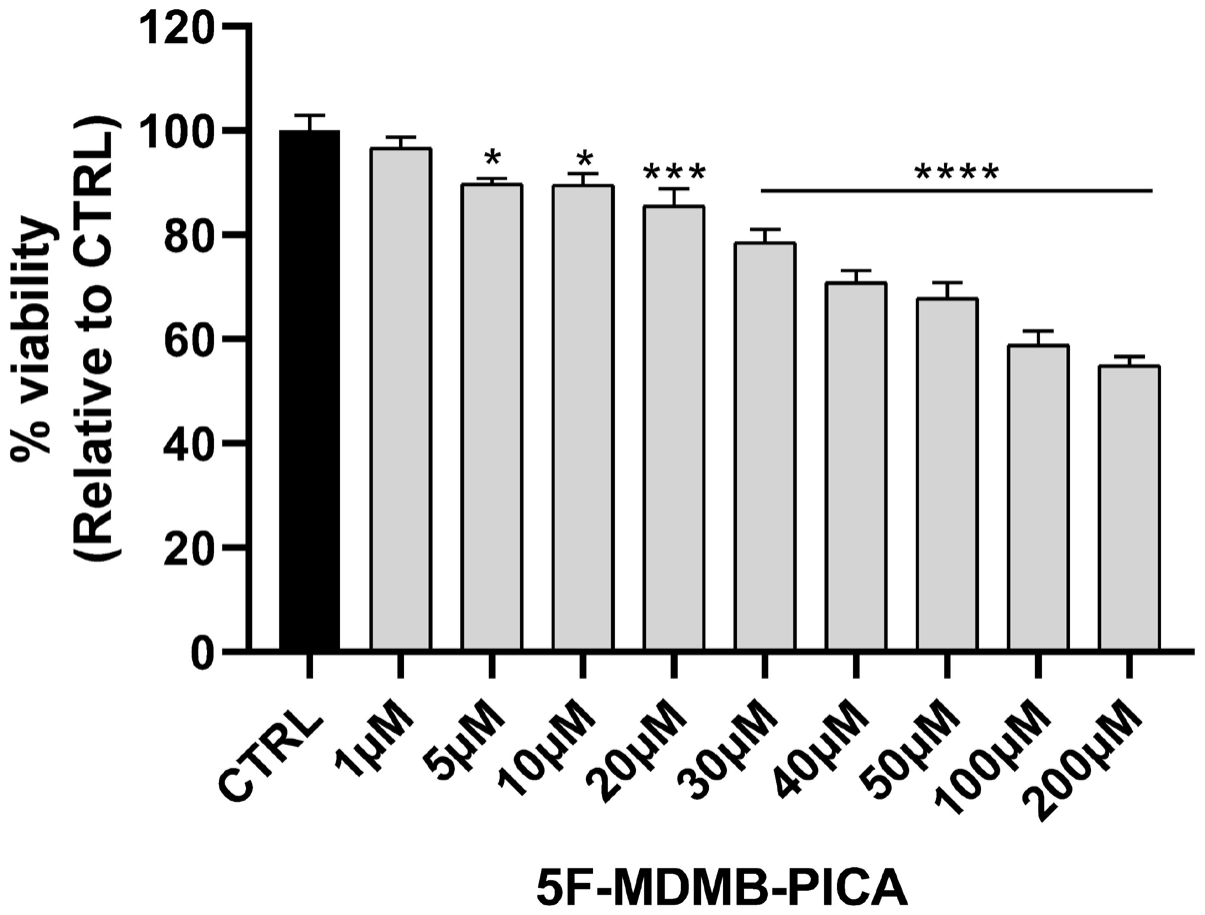
**Effects of 5F-MDMB-PICA on U87-MG cell viability**. Cells were treated with different concentrations of 5F-MDMB-PICA for 24 h. Cytotoxicity was evaluated using an MTT assay at 570 nm. Data shown as the mean ± SEM. *** P < 0.001 and **** P < 0.0001

U87-MG cells exposed to a wide range of *5F-MDMB-PICA* concentrations (1-200 μM) for 24 h showed a significant concentration-dependent reduction in cell viability. The lowest concentration reduced the viability of U87-MG cells was 20 μM (~15%, P < 0.001). The most significant reduction in U87-MG cell viability was observed at 50 μM (~35%, P < 0.0001), 100 (~40%, P < 0.0001), and 200 μM (~50%, P < 0.0001) of 5F-MDMB-PICA. Therefore, these concentrations were selected for the subsequent experiments **(Fig. 2)**.

### 3.2. 5F-MDMB-PICA Induced Oxidative Stress in U87-MG Cells

**Figure 3:**
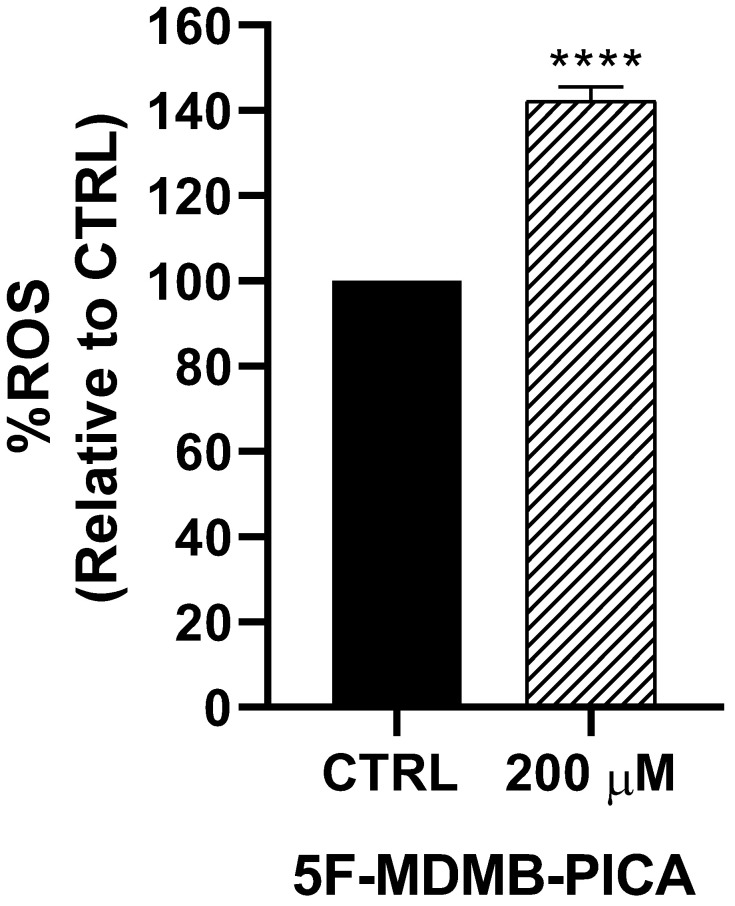
**Effects of 5F-MDMB-PICA on the production of reactive oxygen species in U87-MG cells**. Cells were treated with 200 µM 5F-MDMB-PICA for 4 h. ROS generation was evaluated using the DCFDA assay at excitation and emission wavelengths of 485 and 535 nm, respectively. Data are shown as the mean ± SEM. **** P < 0.0001

Exposure of U87-MG cells to 200 μM 5F-MDMB-PICA for 4 h significantly increased ROS production (~42%, P < 0.0001) compared with the control group** (Fig. 3)**.

### 3.3. 5F-MDMB-PICA Disrupted the Mitochondrial Membrane Potential in U87-MG Cells

**Figure 4:**
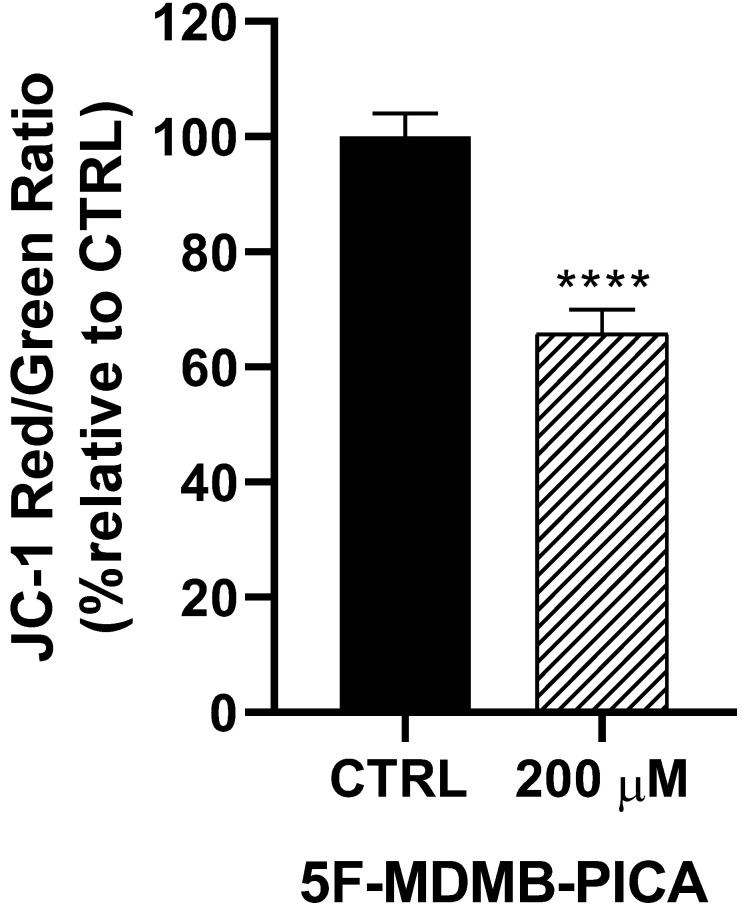
**Effects of 5F-MDMB-PICA on the mitochondrial membrane potential of U87-MG cells**. Cells were treated with 200 µM 5F-MDMB-PICA for 24 h. The effect of 5F-MDMB-PICA on *Δψm* was evaluated using the JC-1 assay at excitation and emission wavelengths of (530 or 590 nm, respectively, for the RED oligomers and at 485 or 528 nm for the GREEN monomers. Data are expressed as the mean ± SEM. * P < 0.05, ** P < 0.01, and **** P < 0.0001

Treating U87-MG cells with 200 μM of 5F-MDMB-PICA for 24 h reduced the mitochondrial membrane potential (Δψm) significantly (34%, P < 0.0001), compared to the control group **(Fig. 4)**.

### 3.4. 5F-MDMB-PICA Reduced U87-MG Cell Migration

**Figure 5:**
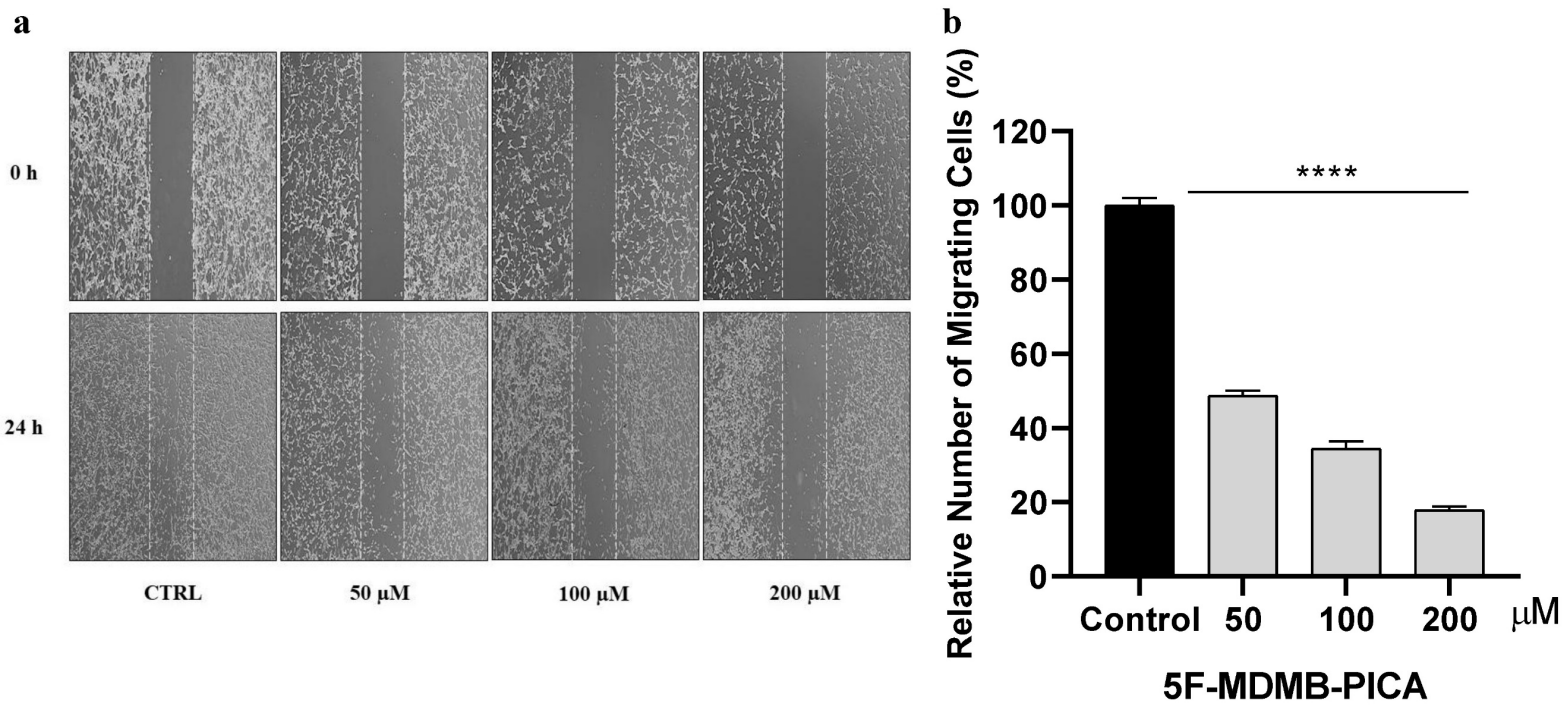
**Effect of 5F-MDMB-PICA on U87-MG cell migration.** Cells were treated with different concentrations of 5F-MDMB-PICA for 24 h. (**a**) Representative photomicrograph of U87-MG migration covering the scratch wound area after 24 h of incubation with various concentrations of 5F-MDMB-PICA compared with the control group. (b) The bar graph shows the number of migrated U87-MG cells compared to the control group. Data are expressed as the mean ± SEM. **** P < 0.0001

The migration of U87-MG cells was impaired in a concentration-dependent manner following 24 h treatment with 50, 100, and 200 μM 5F-MDMB-PICA. U87-MG cell migration was significantly inhibited by ~51% (P < 0.0001), 65% (P < 0.0001), and 80% (P < 0.0001) at concentrations of 50, 100, and 200 μM, respectively **(Fig. 5)**.

### 3.5. 5F-MDMB-PICA Induced Apoptosis and Necrosis in U87-MG Cells

**Figure 6:**
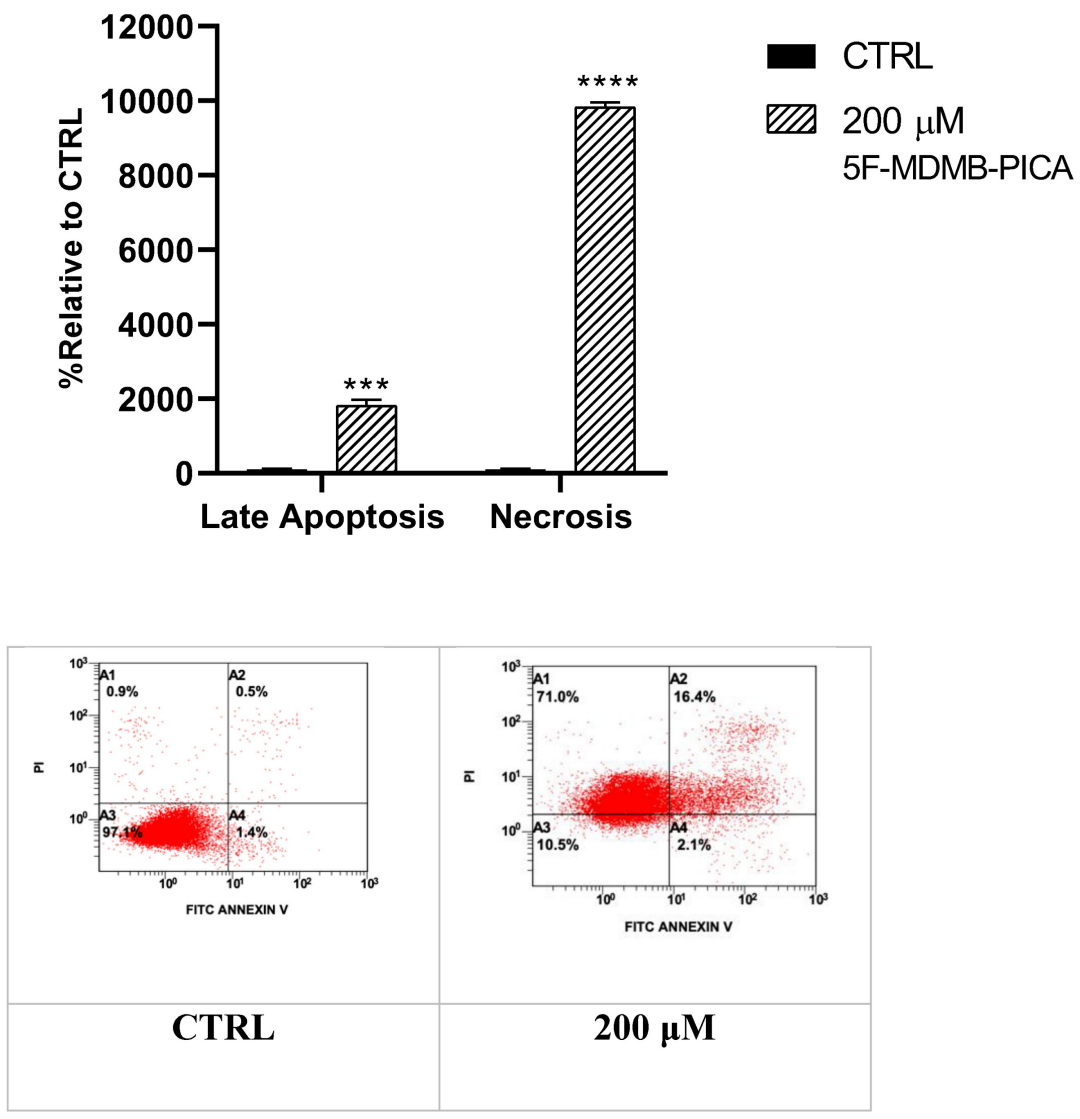
** Effects of 5F-MDMB-PICA on the apoptosis and necrosis of U87-MG cells**. Cells were treated with 200 µM 5F-MDMB-PICA for 24 h. Apoptosis and necrosis levels were evaluated using annexin V- FITC/PI flow cytometry assay. Data shown as the mean ± SEM. *** P < 0.001 and **** P < 0.0001.

Treating U87-MG cells with 200 μM 5F-MDMB-PICA for 24 h significantly increased apoptosis levels (11-fold, P < 0.001) compared to the control group. Interestingly, the necrosis in cells treated with 200 μM 5F-MDMB-PICA was dramatically induced by approximately 90 times (P < 0.0001) compared with the control group **(Fig. 6)**.

### 3.6. 5F-MDMB-PICA Modulates the Expression of Pro-Apoptotic and Inflammatory Genes in U87-MG Cells

**Figure 7:**
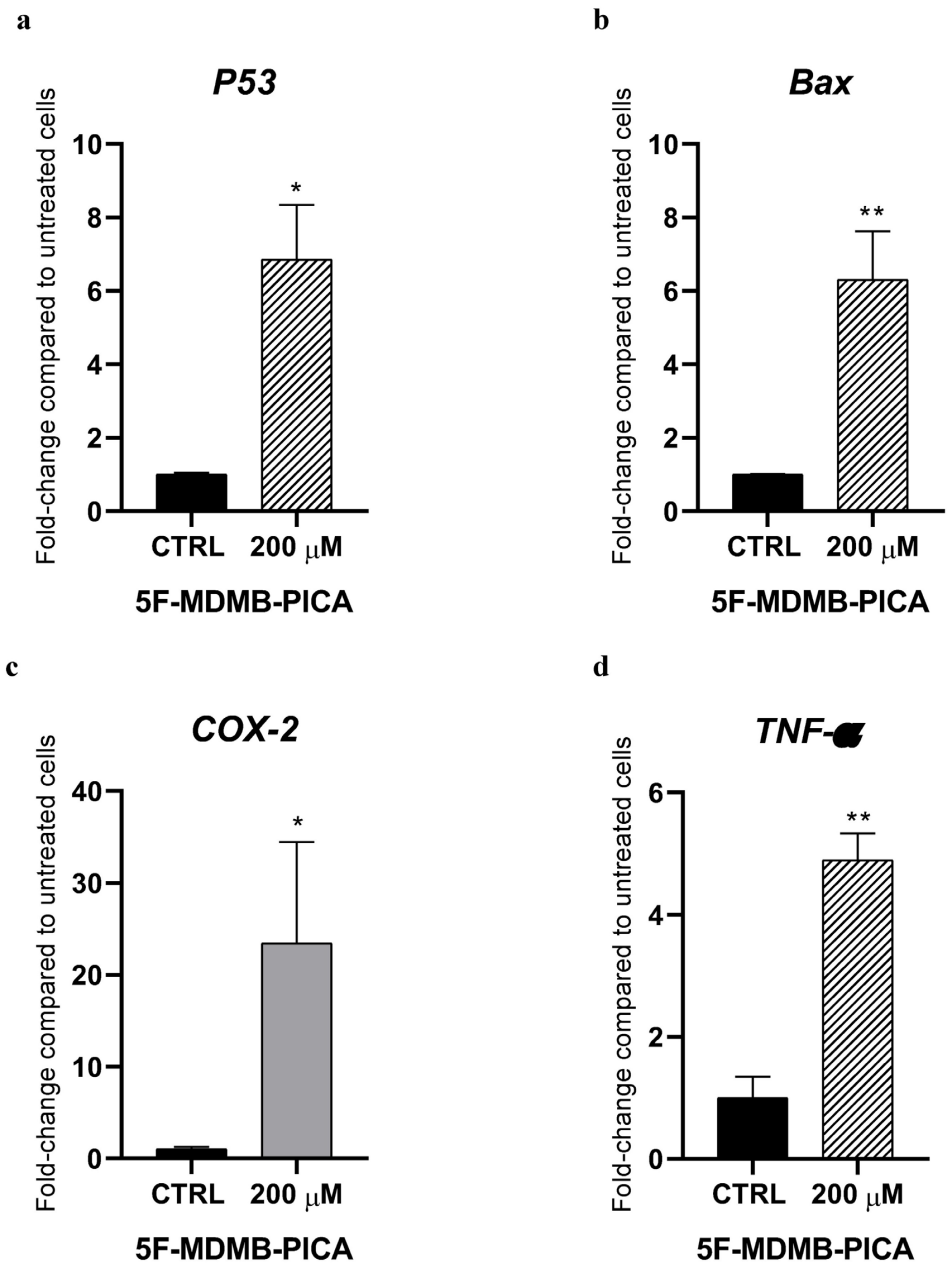
** Effects of 5F-MDMB-PICA on the expression of apoptotic and inflammatory genes in U87-MG cells**. Cells were treated with 200 µM 5F-MDMB-PICA for 24 h. *P53* (a), *Bax* (b), *COX-2* (c), and *TNF- α* (d) genes expression were evaluated using qPCR. Data are shown as fold change compared to control group and expressed as the mean ± SEM. * P < 0.05 and ** P < 0.01

Treating U87-MG cells with 200 μM 5F-MDMB-PICA significantly increased the expression of the pro-apoptotic gene *P53* (6-folds) compared with the control group (**Fig. 7a**). Similarly, expression of the pro-apoptotic gene *Bax* increased significantly following treatment with 200 μM 5F-MDMB-PICA (6-folds) compared with the control group (**Fig. 7b**). Moreover, the expression of the pro-inflammatory cyclooxygenase 2 (*COX-2*) increased significantly in U87-MG cells treated with 200 μM 5F-MDMB-PICA (20-folds) compared with the control group (**Fig. 7c**). Finally, the expression of pro-inflammatory tumor necrosis factor alpha (*TNF-α*) increased significantly in cells treated with 200 μM (4.8-folds, P < 0.01) compared with the control group (**Fig. 7d**).

### 3.7. Analysis of Protein-Ligand Interaction Through Molecular Docking

**Figure 8:**
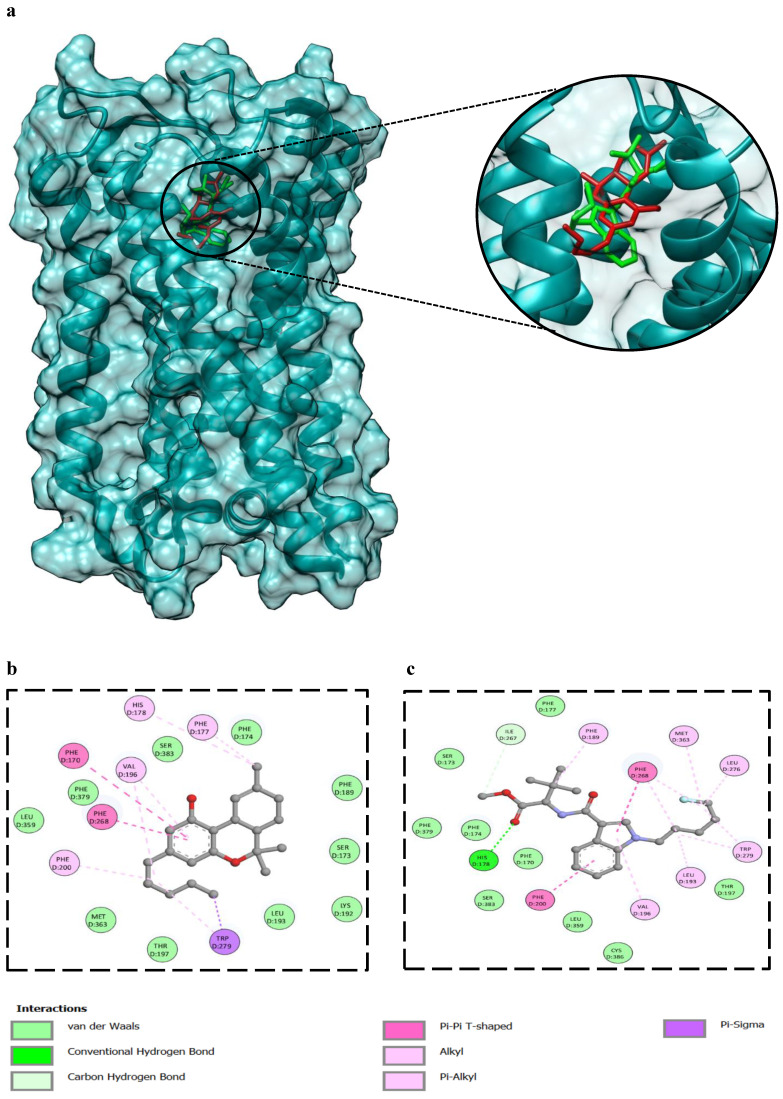
** Molecular docking of compounds against CNR1.** (a) The 3D binding configuration of THC (Red), 5F-MDMB-PICA (Green). Both ligands occupied similar spots of our protein target. The molecular interaction fingerprints in 2D showed that atoms of THC (b) and 5F-MDMB-PICA (c) interacted with amino acid residues important for binding with CNR1.

The binding energy of Δ9-THC against the CB1 receptor (-10.1 kcal/mol) is comparable to 5F-MDMB-PICA (-8.5 kcal/mol). 2D interaction analysis, showed several residues of the CB1 binding pocket were highlighted as key contributors to Δ9-THC and 5F-MDMB-PICA (**Fig. 8b and c**). Among them, Phe200 in both ligands, which has been experimentally validated as essential for ligand binding, where mutational studies have shown that alterations at these positions significantly reduce affinity and impair receptor activity 23. Ser383 was also identified in the Δ9-THC and 5F-MDMB-PICA interaction; this residue plays an important role in modulating binding to CB1 23. Furthermore, the aromatic cluster, including Phe170 and Phe174, forms a stabilizing microenvironment for CB1 24. Additional residues, such as Val196 and Leu193, were found to orient around the ligand, thereby stabilizing CNR1 24.

## 4. Discussion

To the best of our knowledge, our study is among the first to investigate the cytotoxic effects of 5F-MDMB-PICA on astrocyte-like U87-MG cells, which serve as a well-established model for examining the effects of both toxic and non-toxic compounds on brain tissue 25. Additionally, this cellular model (U87-MG) has been found to express the cannabinoid receptor (CB1) 26. The cytotoxicity of this substance, along with its effects on oxidative stress, mitochondrial function, apoptosis, and inflammation, was evaluated. In this study, 5F-MDMB-PICA induced cytotoxicity in U87-MG cells in a significant, concentration-dependent manner. These results are consistent with those observed in another indole analog of SCs (JWH-018). Sezer et al. 27 showed that JWH-018 significantly decreased the viability of neuron-like SHSY5Y cells. In addition, it induced oxidative stress by decreasing the concentration of the antioxidant glutathione and increasing that of the pro-oxidant malondialdehyde 27. In another study, the cytotoxic effects of SCs were associated with apoptosis. The harmful effects of SCs have been shown to be mediated by activation of CB-1R, but not of CB-2R 28.

The induction of apoptosis, oxidative stress, and mitochondrial dysfunction biomarkers is associated with numerous neurological disorders and neurodegenerative diseases. Furthermore, it has been implicated in the clinical manifestations and pathophysiology of these diseases, including neuronal loss and behavioral changes 29-31. Our study confirmed that 5F-MDMB-PICA-induced oxidative stress is concentration-dependent. The correlation among SCs, oxidative stress, and neurotoxicity is well established 27,32. Activation of the CB1 receptor has been linked to SC-mediated neurotoxicity and oxidative stress 32. Furthermore, our study demonstrated that 5F-MDMB-PICA induced mitochondrial dysfunction by decreasing Δψm, an indicator of cytotoxicity and apoptosis. Interestingly, this finding is consistent with previous reports in confirming a correlation between SCs and mitochondrial dysfunction 33. These results suggest that 5F-MBMB-PICA exposure can lead to harmful manifestations, including aggression, hallucinations, changes in perception and mood, memory impairment, and panic attacks, by inducing neurotoxicity, oxidative stress, and mitochondrial dysfunction.

We further used flow cytometry to confirm whether 5F-MDMB-PICA significantly induces apoptosis and necrosis in U87-MG cells. Our findings demonstrated that the cytotoxic effect of 5F-MDMB-PICA is associated with the induction of apoptosis and necrosis. Funada et al. 34 have shown that SC-induced neuronal apoptosis occurs via CB1 receptor-mediated caspase-3-dependent pathways. Another study has shown that SCs induced apoptosis, metabolic alterations, and morphological changes in astrocytes 35. Necrosis has also been reported in association with the use of synthetic cannabinoids 36. Furthermore, our PCR analyses indicated that 5F-MDMB-PICA enhances the expression of pro-apoptotic and pro-inflammatory genes, specifically Bax, P53, COX-2, and TNF-α. These results provide insight into the possible molecular mechanisms underlying the harmful manifestations of 5F-MDMB-PICA.

Our results correlate with the previous studies that reported the cytotoxic effects of SCs on the CNS 34, 37. Neuroinflammation and alterations in cannabinoid receptor signaling have been linked to cannabinoid addiction 38. Furthermore, induction of inflammation by SCs has been associated with the activation of CB1 receptors 39, 40. Notably, our recently published study revealed that 5F-MDMB-PICA significantly reduced the expression of glutamate transporters in U87-MG cells, including GLT-1, GLAST, and xCT. This suggests that 5F-MDMB-PICA may induce cytotoxicity in U87-MG cells via excitotoxic mechanisms 41. Finally, migration experiments demonstrated that 5F-MDMB-PICA significantly inhibited U87-MG cell migration in a concentration-dependent manner. This finding is consistent with previous studies showing that cannabinoids can inhibit proliferation and invasion in U87-MG cells. These effects have been linked to those of cannabinoids on ERK1/2 and Akt 42. Taken together, our study demonstrated the cytotoxic effects of 5F-MDMB-PICA on the CNS. Additionally, these findings provide encouraging data from an oncological perspective, potentially paving the way for future research into the impact of 5F-MDMB-PICA on different cancer cell types. Notably, the anti-tumor activities of SCs have been the focus of considerable research in recent years 43,44. Our study has, for the first time, demonstrated that Δ9-THC and 5F-MDMB-PICA exhibit overlapping binding residues (Phe200 and Ser383) within the CB-1 receptor pocket. Indeed, since both compounds engage a shared binding site on the CB-1 receptor, this may help elucidate the similarities in their cytotoxic effect patterns. Future studies are warranted to further validate our molecular docking findings through molecular dynamics simulations and binding free energy calculations, which would provide deeper insight into the stability of the ligand-receptor complex and the reliability of the predicted binding interactions. Although the present study demonstrates cytotoxic effects of 5F-MDMB-PICA in a human glioblastoma cell line, further investigation in relevant *in vivo* glioblastoma models, followed by clinical studies, is required to assess pharmacokinetics, blood-brain barrier penetration, and translational relevance. Moreover, studies in animal models would enable correlation of the observed cellular effects with *in vivo* and behavioral outcomes associated with 5F-MDMB-PICA exposure. Our study is the first to investigate the effects of 5F-MDMB-PICA on astrocyte-like U87-MG cells.

## 5. Conclusions

**Figure 9:**
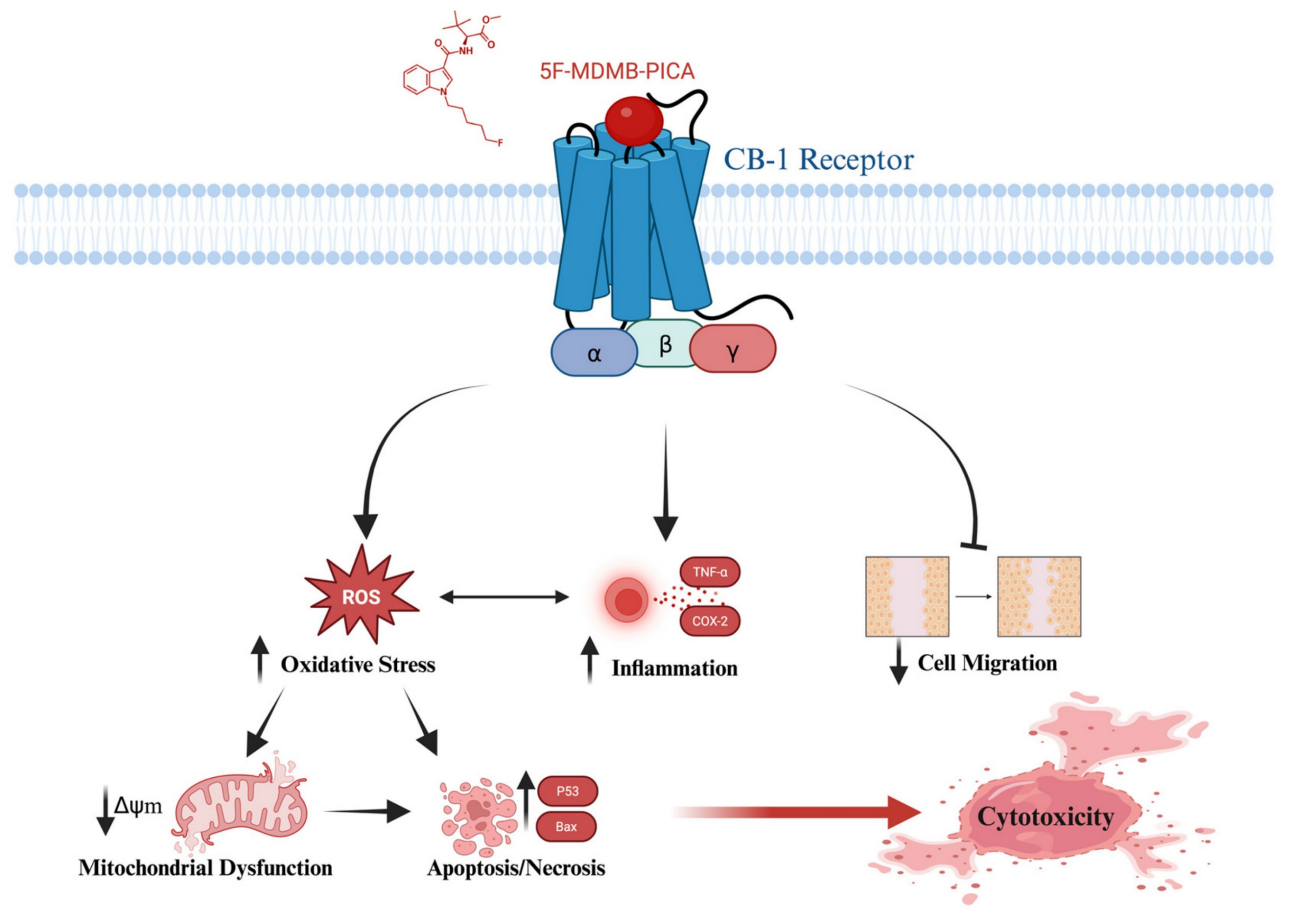
** Graphical abstract of the proposed cytotoxic mechanism for the synthetic cannabinoid, 5F-MDMB-PICA on U87-MG cells**. Cannabinoid Receptor 1 (CB-1); Reactive Oxygen Species (ROS); Tumor Necrosis Factor- alpha (*TNF-α*); Cyclooxygenase-2 (*COX-2*); Mitochondrial Membrane Potential (Δψm); Cellular Tumor suppressor gene (*P53*); Bcl-2 associated X (*Bax*).

This study provides compelling evidence that 5F-MDMB-PICA induces neurotoxicity through apoptosis, oxidative stress, inflammation, and mitochondrial dysfunction in astrocyte-like U87-MG cells. Also, this study has shown that both Δ9-THC and 5F-MDMB-PICA bind to a shared site on the CB-1 receptor. These findings may clarify the mechanisms underlying the harmful effects associated with the consumption of this synthetic cannabinoid **(Fig. 9)**, providing valuable insights into its detrimental impact on health. To the best of our knowledge, our research is the first to present toxicological data regarding the impact and the cytotoxicity mechanisms of 5F-MDMB-PICA on the CNS.

## Figures and Tables

**Table 1:** Forward and reverse primer sequences

No.	Gene Symbol	Forward Primer Sequence	Reverse Primer Sequence
1	*Bax*	5′-GTTTCATCCAGGATCGAGCAG-3′	5′-CATCTTCTTCCAGATGGTGA-3′
2	*P53*	5′-CCCCTCCTGGCCCCTGTCATCTTC-3′	5′-GCAGCGCCTCACAACCTCCGTCAT-3′
3	*TNF-⍺*	5′-CTCTTCTGCCTGCACTTTG-3′	5′-ATGGGCTACAGGCTTGTCACTC-3′
4	*COX-2*	5′-CCCTTGGGTGTCAAAGGTAA-3′	5′-GCCCTCGCTTATGATCTGTC-3′
5	*GAPDH*	5′-GCCAAGGTCATCCATGACAACT-3′	5′-GAGGGGCCATCCACAGTCTT-3′

## Data Availability

All data presented in this study are available upon reasonable request from the corresponding authors.
